# Patterned Liquid Crystal Polymer Thin Films Improved Energy Conversion Efficiency at High Incident Angles for Photovoltaic Cells

**DOI:** 10.3390/polym16101358

**Published:** 2024-05-10

**Authors:** Gwomei Wu

**Affiliations:** Institute of Electro-Optical Engineering, Chang Gung University, Chang Gung Memorial Hospital, Taoyuan 333, Taiwan; wu@mail.cgu.edu.tw; Tel.: +886-3211-8800

**Keywords:** photovoltaic cell, polymer thin film, patterned, energy conversion efficiency, incident angle

## Abstract

In this report, micro-patterned silicon semiconductor photovoltaic cells have been proposed to improve the efficiency in various incident sunlight angles, using homeotropic liquid crystal polymers. The anisotropic liquid crystal precursor solution based on a reactive mesogen has good flowing characteristics. It can be evenly coated on the silicon solar cells’ surface by a conventional spreading technique, such as spin coating. Once cured, the polymers exhibit asymmetric transmittance properties. The optical retardation characteristics of the coated polymer films can be eventually determined by the applicable coating and curing parameters during the processes. The birefringence of light then influences the optical path and the divergence of any encountered sunlight. This allows more photons to enter the active semiconductor layers for optical absorption, resulting in an increase in the photon-to-electron conversion, and thus improving the photovoltaic cell efficiency. This new design is straightforward and could allow various patterns to be created for scientific development. The experimental results have evidenced that the energy conversion efficiency could be improved by 2–3% for the silicon photovoltaic cells, under direct sunlight or at no inclination, when the liquid crystal polymer precursor solution is prepared at 5%. In addition, the efficiency could be much more significantly improved to 14–16% when the angle is inclined to 45°. The unique patterned liquid crystal polymer thin films provide enhanced energy conversion efficiency for silicon photovoltaic cells. The design could be further evaluated for other solar cell applications.

## 1. Introduction

Reliable energy is essential in providing the sustainable economic and social development much needed for modern human life [1,2,3]. Due to the rising cost of natural energy resources and the growing need for environmental protection, the demand for alternative power generation technologies is becoming increasingly important. Recently, global warming and the issues related to climate change have become increasingly serious problems. The excessive emission of greenhouse gases, such as carbon dioxide and sulfur oxides, from the combustion of fossil fuels and coals is likely the principal cause. Among the renewable energy alternatives, solar energy has the potential to meet the growing electricity demand [4,5,6,7]. It can also help to relieve stress from the shrinking fossil resources and global carbon dioxide emissions.

Numerous research efforts have focused on sustainable energy production based on the capture and conversion of sunlight into more useful forms, such as electricity and heat [8,9]. The subsequent storage of the acquired energy is also gaining importance, since this would allow supply for future uses. Photovoltaic cells are such attractive devices that can capture photons and convert them directly into electric energy in a clean and sustainable way. However, silicon photovoltaic cells account for less than 1% of the total energy production. This low market contribution is mainly caused by the relatively high cost per kilowatt-hour of photovoltaic cells [10]. There are two strategies used to reduce the energy production cost. One is lowering the material and device cost, and the other one is increasing the energy conversion efficiency. The important purpose of this study is therefore to develop ways to improve energy conversion efficiency, and then to lower the cost per kilowatt-hour of photovoltaic cells.

Silicon photovoltaic cells with a higher photon-to-electron conversion efficiency have evolved substantially over the last few years, mainly due to concerns regarding natural resources and environmental protection. On top of the advances achieved in semiconductor technology, the creative design of the optical system to minimize unnecessary photon reflection in order to improve the incoming sunlight’s optical absorption has become quite desirable for continued success in this highly anticipated and promising industry [11,12]. The refractive index of single crystalline silicon is 6.0–3.5 at the sunlight wavelength range of 400–1100 nm. The optical loss of reflection can be more than one half in the shorter wavelength side, and one third in the longer wavelength side. On the other hand, natural sunlight changes its incident angle during the day, and the reflection inevitably increases with the increased angle [13]. A new design to track the sunlight could surely increase the corresponding energy conversion efficiency to almost the values of a direct sunlight system. But this creates additional costs in hardware assembly, and a fraction of the valuable electrical energy would be wasted again for this kind of operation. A common method has been to texture the solar cell surface by wet chemical etching, which utilizes a corrosive acid or an alkaline solution to carve the exterior surface of the silicon. The etched pyramidal structures have been reported to redirect some of the reflected photons for subsequent optical absorption. This approach also improves some solar cells’ energy conversion efficiency [14].

With the rapid advancements in photo patterning processing, even periodic nanostructures with periods that are smaller than the incident light wavelength can be precisely implemented [15]. There has been increased demand for rapid and massive techniques for large-area processing. In the literature, Yu et al. reported the creation of subwavelength structures by holographic lithography and dry etching [16]. Lee et al. revealed the broadband antireflection improvements enabled by small-size gratings for crystalline silicon photovoltaic cells [17]. Shi et al. used a sol–gel technique to provide several layers of antireflection coatings, also for a silicon solar cell system [18]. Mohseni et al. studied nanosphere photolithography on different photoresist materials for large-area applications. They also demonstrated some good nanohole array results using micro-spheres [19]. Kim et al. presented the use of nanosphere lithography with SiO_2_ in a benzocyclobutene layer, and the reflectance was effectively reduced [20]. The commercial applications will still rely on the successful development of large-scale and reliable manufacturing processes with enhanced power conversion efficiency [21,22]. It remains a technical challenge to distribute nanoparticles very uniformly on the surface of the silicon substrate. The nanoparticle size can be very small in the range of 200–1000 nm. The subsequent curing solution can be unevenly distributed among those nanoparticles by all the conventional coating techniques. The high processing cost surely poses an additional limitation.

It has been shown that anisotropic polymer thin films with optical birefringence can be designed using reactive mesogen chemistry [23,24]. We are therefore interested in developing patterned homeotropic polymer thin films on silicon photovoltaic cells that exhibit reduced reflection characteristics, so that the silicon surface texturing effect can be put into practice while the corrosive chemical etching method is avoided. The effective angle of incident sunlight can be greatly improved. The optical retardation characteristics of the liquid crystal polymer thin films can be determined by the adequate coating and curing parameters. The liquid crystal polymer reactive mesogen precursor has good flowing properties. It can be evenly coated on the silicon surface by a low-cost conventional technique, such as spin coating. The unique design is straightforward and can create different patterns for investigation. Three photo-masks are prepared for parallel UV (ultraviolet) curing exposure, with periodical arrays in a triangle, square and honeycomb. The homeotropic liquid crystal polymer thin film photolithography technique is straightforward and is less expensive than electron beam lithography or focused ion beam technology. The surface patterned photovoltaic cells are investigated using the Solar Simulator Model XES-151S with the spectral source AM 1.5 G at different sunlight incident angles. The optical reflectance is measured using Solar Cell Scan100 using QE (quantum efficiency measurement system) and IPCE (monochromatic incident photon-to-electron conversion efficiency). The birefringence of light, due to the homeotropic polymer thin film’s asymmetric optical characteristics, changes the optical path and the divergence of any encountered sunlight photons. This allows more photons to enter the active semiconductor layers for optical absorption and results in an increase in photon-to-electron conversion. The photovoltaic cell efficiency could thus be improved. The aim of this article is to present a new method that can effectively reduce multiple angle reflection for incident sunlight and enhance the silicon photovoltaic cell energy conversion efficiency. The effects of the polymer thin film precursor concentration and the designed patterns on the silicon photovoltaic cell efficiency enhancement are carefully investigated. Once the successful development of the new patterned polymer system is achievable, it could pave the way for many new ideas for all types of photovoltaic cells.

## 2. Materials and Methods

The silicon photovoltaic cell experimental samples (STR-10) were received from SolarTech, Inc. (Hsinchu, Taiwan). They were cleaned in acetone, isopropyl alcohol, and de-ionized water. The silicon samples had been cut into a square shape of 10 × 10 mm^2^ in size. The organic precursor was composed of a rigid-rod liquid crystal reactive monomer with vertical alignment characteristics (Merck 3015, Darmstadt, Germany). It could be diluted to various concentrations of 1–10% by weight using propylene glycol monomethyl ether acetate (PGMEA, Aldrich, St. Louis, MO, USA). The room-temperature viscosity was around 2.3~2.8 centi-Poise, mainly depending on the monomer concentration [23,25]. For the polymer thin film fabrication process, the viscous precursor solution was evenly spread onto the silicon photovoltaic cell specimens using Apisc’s spin-coating unit (SP-M1-S, Taoyuan, Taiwan) in two steps. The first step was at 500 rpm (round per minute) for 10 s and then the second step was at 3000 rpm for another 30 s. A soft bake at 100 °C was carried out for 80 s to drive out the excessive solvent. A further UV light curing step was carried out using an 8-Watt light source (wavelength 365 nm) under nitrogen purge for 3.5 min. The UV light power density was nominally calculated to be around 8 mW/cm^2^. The solar energy’s opto-electrical conversion efficiency for the coated semiconductor photovoltaic cell samples was evaluated by San-Ei Electronic Co.’s solar simulator (Model XES-151S, Osaka, Japan) to reveal the suitable concentration for the precursor formulation.

**Figure 1:**
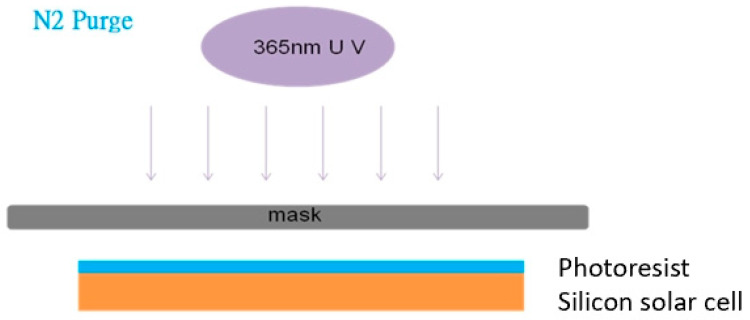
UV light exposure process for the patterned homeotropic liquid crystal polymer thin films on silicon photovoltaic cells with photo masks.

**Figure 2:**
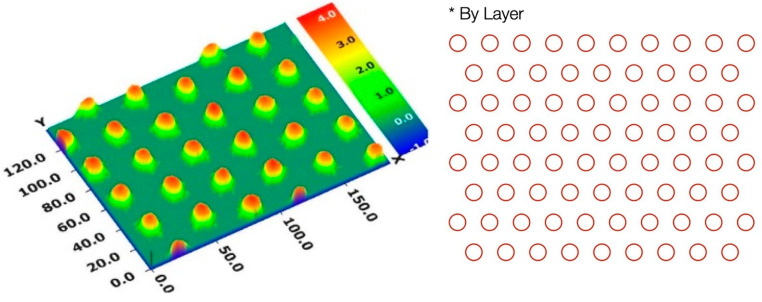
Three-dimensional optical micrograph of a patterned silicon photovoltaic cell. The corresponding mask design with the periodical triangular array is also shown.

The patterned silicon photovoltaic cells were mainly prepared from the 5% reactive mesogen polymer precursor solution. Three patterning masks had been selected for the photo-lithography process. The periodical arrays were prepared in a triangle, square or honeycomb. After the spin coating of the polymer precursor solution, the coated samples were cured by parallel UV light exposure (see Figure 1). The section that was hampered by a photo mask was removed by dipping it in an organic solvent after the UV exposure, so that the array patterns would be correctly transferred to the silicon cells. Eventually, the samples were heated at 180 °C for 1 h to set the structure. An example of the surface morphology of the patterned homeotropic polymer thin films on silicon photovoltaic cells with the triangle array is shown in Figure 2.

We studied three different incident light angles, including 0°, 30° and 45°. All the measurements were carried out at the ambient temperature. The incident light angle was referred to as the angle between the incident source light and the direction of the imaginary normal line from the photovoltaic cell sample surface. The measurement experiments were conducted by rotating the photovoltaic cell samples on an optical stage to the specified orientation before the measurement. A protractor was used to record the incident angle. All the patterned silicon photovoltaic cells were evaluated by a solar simulator using Model XES-151S with the spectral source AM 1.5G (IEC 904-9 Class B). This employed a 150 W Xenon short-arc lamp. The irradiance uniformity was within 5% and the temporal instability of the irradiance was less than 1%. On the other hand, the Solar Cell Scan 100 system (Zolix, Taipei, Taiwan) was employed to examine the photovoltaic cell samples’ reflectivity characteristics in the wavelength range of 400 up to 1000 nm. This is consistent with the IEC 60904-8 international standards, with a double-light-path intensity monitoring function. The testing cell area was up to 100 × 100 mm^2^. The simulated sunlight opto-electrical energy conversion efficiency results could then be investigated for designing new cell structures using different processing variables and tools.

## 3. Results and Discussion

**Figure 3:**
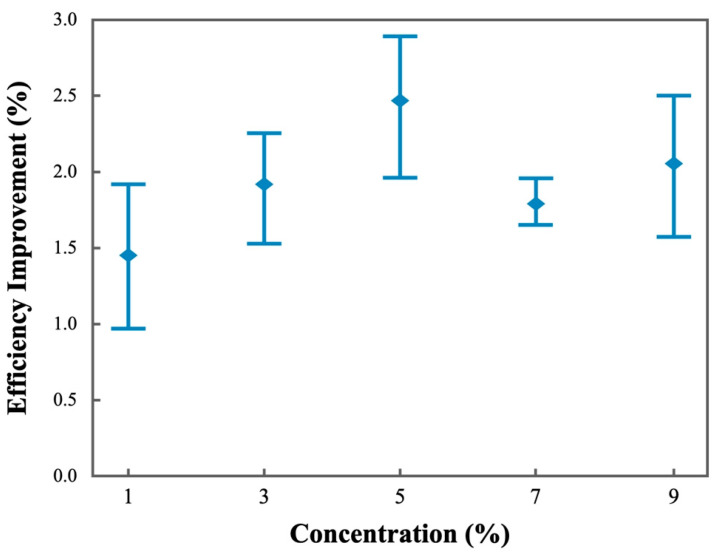
Energy conversion efficiency improvement results for the homeotropic polymer thin-film-coated photovoltaic cell samples.

The 365 nm UV light exposure created homeotropically aligned anisotropic liquid crystal polymer thin film structures with birefringence on the silicon photovoltaic cells. It improved the solar cell energy conversion efficiency by guiding additional lights in the perpendicular direction, which allowed more photons to be captured and absorbed by the active semiconductor layers in the silicon photovoltaic cells. The reflection and the unnecessary scattering could be thus slightly reduced. Figure 3 shows the measured efficiency improvement for the silicon photovoltaic cell samples prepared from the precursor solutions with various concentrations. The corresponding solar cell characteristic parameters are also summarized in Table 1. The control sample represents the one with no polymer coating. The homeotropic liquid crystal thin film thickness was estimated to be 1–5 μm, depending on the concentration of the prepared precursor solution and the curing parameters. The experimental results suggested that the 5% precursor concentration coated sample exhibited a relatively higher efficiency improvement. It was thus recommended as a suitable choice for our applications. The energy conversion efficiency could be improved by about 2.5%. It has been noted that a sample with a much lower solution concentration would become too thin due to the very low solid content. A much thinner coating layer was prepared. The corresponding photo initiator was also highly diluted in the solution, which could make the UV light curing process difficult to control. On the other hand, the solution would become more viscous if the precursor concentration was much higher. This could make it more difficult to form a homogeneous thin film coating on the silicon photovoltaic cells. A thicker coating layer may inevitably absorb more photons, thereby decreasing the light transmittance. Sometimes, the coating simply became partially opaque, due to the inclusion of small air bubbles. In addition, the 5% sample showed a higher improvement of 3–4% at the inclined incident angle of 45° from sunlight. The birefringence for light then controls the optical path and the propagation of the incoming photons in the films. This would allow more photons to be captured in the active semiconductor structure and therefore could increase the desirable photon-to-electron energy conversion effects.

**Figure 4:**
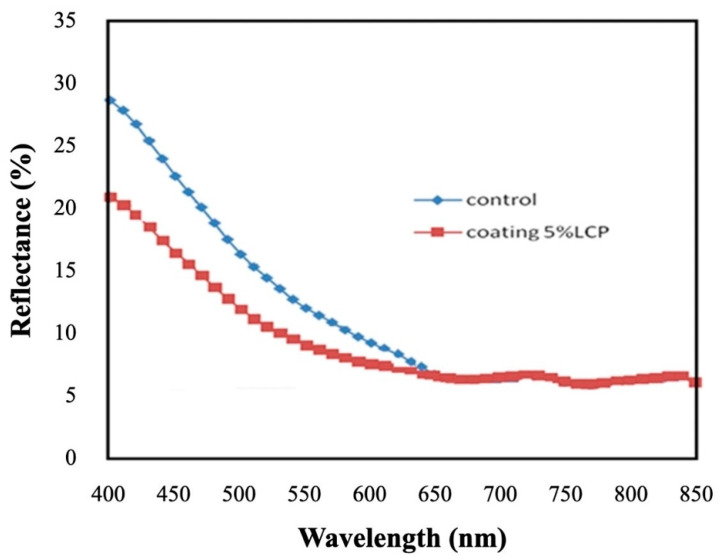
Reflectivity spectra for the homeotropic polymer thin film and the control photovoltaic cell samples using the Solar Cell Scan 100 system.

**Figure 5:**
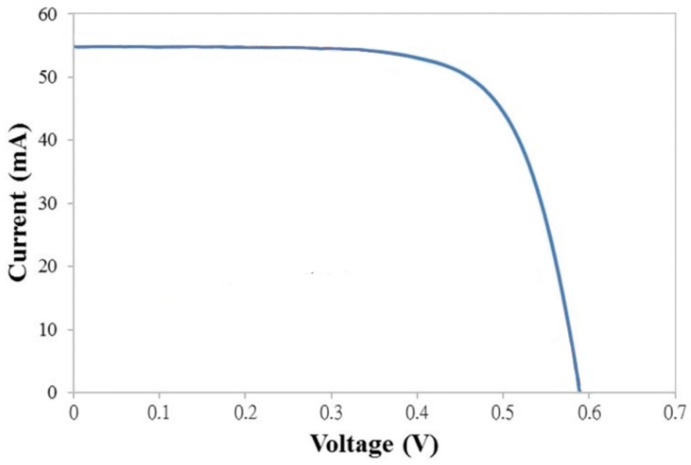
Current–voltage (I–V) characteristics of the polymer-coated silicon photovoltaic cell.

Figure 4 displays the reflectivity spectra for the homeotropic polymer thin-film-coated and control (without the polymer coating) silicon photovoltaic cell samples. The measuring wavelength range is from 400 to 850 nm, and the Solar Cell Scan 100 system is used. The data show that the 5% polymer precursor sample had a lower reflectivity, specifically in the shorter wavelength range of 400–600 nm. Antireflection coating designs have been reported to improve the photoelectric conversion efficiency of photovoltaic cells [26,27]. For example, the reflectivity was reduced from 15.8% to 11.7% at 500 nm. This is in good agreement with the energy conversion efficiency improvement results already described in Figure 3. On the other hand, an example of the current–voltage (I–V) characteristics of the coated silicon photovoltaic cell when a 5% polymer concentration was employed is shown in Figure 5. From the measurement curve, it is clear that the turn-on voltage is about 0.5~0.6 V [28,29].

**Figure 6:**
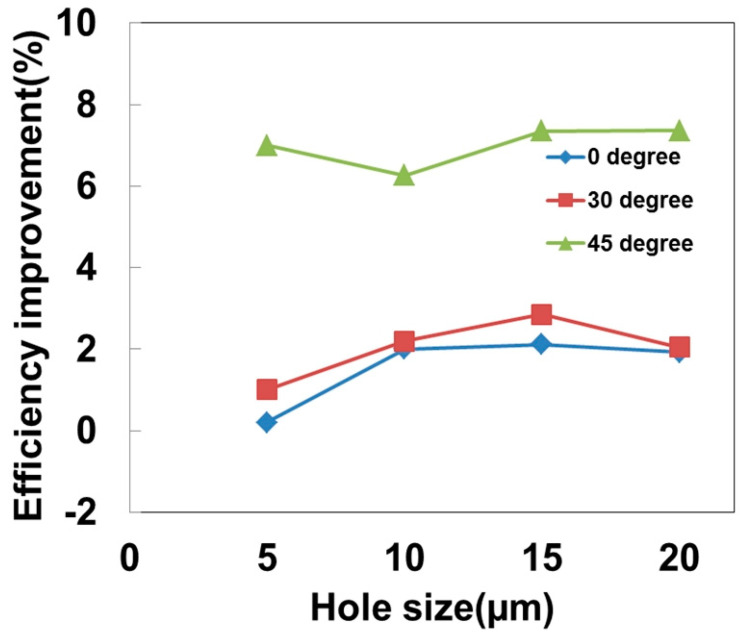
The energy conversion efficiency improvement analysis results from the 5% solution and triangular-array-patterned silicon photovoltaic cells. The improvements are much more evident at the high incident angle of 45°.

All the patterned homeotropic polymer thin films on the silicon photovoltaic cells were prepared using only a 5% precursor solution. Figure 6 shows the energy conversion efficiency improvement results from the triangular array pattern with the different patterning hole diameters. The period, the distance between any neighboring holes, is twice the hole diameter (a/d = 2). After the photolithography process, the homeotropic liquid crystal polymer thin film in the holes, which had been cured by UV exposure, became solid bumps on the silicon photovoltaic cells. It has been suggested that the energy conversion efficiency of the 5% patterned homeotropic polymer thin film silicon photovoltaic cells was mostly improved by a couple of percent. However, it exhibited much higher values of 6–8% when the incident sunlight angle was increased to 45°. The medium patterning hole sizes (10–15 μm) seem to be suitable for a good conversion design, and they are suitable for rapid and massive manufacturing processes in a large area. It has been shown that the energy conversion efficiency of a silicon photovoltaic cell becomes deteriorated when the sunlight incident angle is further increased. This is the reason for the industry utilizing a conventional tracking system to overcome this obstacle, so that the ultimate objective of achieving direct sunlight harvesting becomes possible. However, a tracking system generally increases the cost of hardware assembly. A small portion of the valuable electrical energy would be wasted again for this kind of operation. In our design, the retardation in the optical properties of the homeotropic polymer thin films could exhibit strong anisotropy in the photon transmittance. The patterned polymer thin film structures changed the optical path and the divergence of incoming photons. This would result in more photons entering the active silicon semiconductor structures for photon capture. Therefore, the cell photon-to-electron conversion efficiency could be effectively increased, specifically with the patterned design at a higher incident angle of 45°.

**Figure 7:**
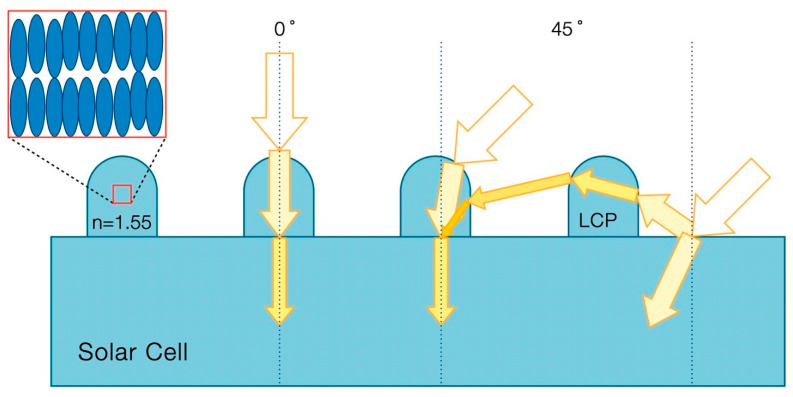
Schematic illustration of the optical paths of incident light under the different inclined angles on a patterned silicon photovoltaic cell. The birefringent polymer thin film bumps redirect the incoming light for more semiconductor absorption.

Figure 7 shows a schematic illustration of the optical paths of incident light under the different inclined angles on a patterned homeotropic polymer thin film silicon photovoltaic cell surface. The birefringence associated with the liquid crystal polymer is 0.12–0.14. The ordinary refractive index (n_o_) of the liquid crystal is nominally 1.53, while the extraordinary refractive index component (n_e_) is slightly higher at about 1.67 for the homeotropically aligned anisotropic polymer thin film bumps [25]. The rigid-rod liquid crystal molecules should be made from a cyclic olefin resin that can exhibit the desired optical retardation. They are all aligned in the vertical orientation due to their polar–nonpolar chain design [30]. The molecular structure arrangement in the vertical alignment fashion of the UV-cured thin film bumps helped in redirecting the incident light toward a position closer to that of direct sunlight at a larger inclined angle (such as 45°). Some of the light beam could become polarized by the asymmetric retardation caused by the polymer bumps. In addition, some of the partially reflected photons from the uncovered area, due to the large refractive index difference between silicon and air (*n* > 3.5 versus *n* = 1.0), were further redirected by the birefringent polymer thin film bumps, thereby making them suitable for semiconductor absorption.

**Figure 8:**
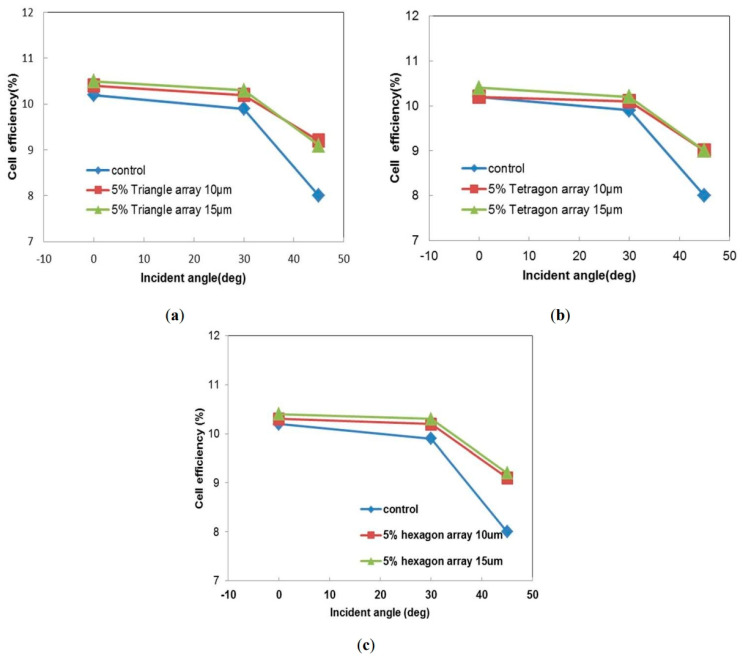
The patterned homeotropic polymer thin film silicon photovoltaic cells’ energy conversion efficiency characteristics from the (**a**) triangle, (**b**) square, and (**c**) honeycomb periodical arrays. The control photovoltaic cell data are also provided for those with no polymer thin film coating.

Three photo-patterning masks were prepared for the UV lithography processes, including the periodical triangle, square and honeycomb arrays. The patterned homeotropic polymer thin film silicon photovoltaic cells’ energy conversion efficiency characteristics were studied using the Solar Simulator Model XES-151S. The efficiency results are displayed in Figure 8. Here, the control silicon photovoltaic cell’s data are provided for that with no polymer thin film structure. Three different sunlight incident angles were used for the cell performance investigation. It has been suggested that the patterned homeotropic polymer thin film silicon photovoltaic cells showed much more significant improvements at the higher inclined incident sunlight angles. In Figure 8a, the cell energy conversion efficiency is slightly increased from 10.2% to 10.4% and 10.5% at the incident angle of 0°, or under direct sunlight, when the triangle array period was 10 μm and 15 μm, respectively. It was increased from 9.9% to 10.2% and 10.3% at the incident angle of 30°, with array periods of 10 μm and 15 μm. However, the photovoltaic cell energy conversion efficiency was much more significantly increased from 8.0% to 9.2% and 9.1% at the inclined sunlight angle of 45°, with periods of 10 μm and 15 μm, respectively. This represents an improvement of 15% in the photon-to-electron energy conversion efficiency. In addition, there has been some concern regarding whether the applied polymer film is durable enough to withstand the atmospheric conditions for a significant period of time. We checked that the silicon solar cell experimental samples showed very stable results after 1 month of storage in an electronic dry box (TDT Dry Tech AX-180) at room temperature. However, a long-term stability and deteriorative evaluation study using a temperature and relative humidity control chamber may be needed for more practical implentation. Some hardness, scratch resistance, roughness, and adhesion to surface tests would provide a more comprehensive view of the material and device design. Although this patterned liquid crystal polymer structure was applied on silicon solar cell substrates in this study, it would be interesting to further apply this on the glass encapsulation material for any photovoltaic cells, including an organic–inorganic hybrid system such as a dye-sensitized solar cell or a perovskite solar cell [31,32].

A similar trend was found in the square and honeycomb-array-patterned homeotropic polymer thin film silicon photovoltaic cells. The square-array-patterned samples exhibited energy conversion efficiency data of 10.2% and 10.4%, with periods of 10 μm and 15 μm, respectively, at the incident sunlight angle of 0°. They became 10.1% and 10.2% at the sunlight angle of 30°. The results were both 9.1% for the incident sunlight angle of 45°. For the honeycomb array cell samples, the energy conversion efficiency at the incident angle of 0° was 10.2% and 10.3% when using periods of 10 μm and 15 μm, respectively. The results were 10.1% and 10.2% at the angle of 30°, and were 9.2% and 9.3% at the sunlight angle of 45°. These small deviations are still within our experimental errors. Nevertheless, they all showed improvements of a couple of percent in the lower sunlight incident angle measurements, while the improvements in the 45° measurements were up to 14–16%. The novel patterned homeotropic polymer thin film structures could thus improve the silicon photovoltaic cell energy conversion efficiency, specifically for the higher incident sunlight angle at 45°. The integrated conversion efficiency would be closer to that of direct sunlight. The proposed patterned homeotropic polymer thin films exhibited unique optical retardation properties, providing high anisotropy characteristics. The precursor solution could be easily applied to form uniform homeotropic thin films on any photovoltaic cell substrates using a conventional spin-coating technique. Therefore, this novel technology can be further developed for rapid and massive manufacturing for large-area processing in the photovoltaic cell industry.

## 4. Conclusions

In this study, we have successfully proposed and demonstrated that patterned homeotropic liquid crystal polymer thin films could effectively improve the energy conversion efficiency of silicon photovoltaic cells, especially at high inclined sunlight incident angles. The novel patterned silicon photovoltaic cells, based on the photolithography design of reactive mesogens, were reported here in the literature for the first time. The homeotropically aligned, anisotropic liquid crystal polymers redirected sunlight from the different incident paths and reduced unwanted reflection. The unique birefringent homeotropic polymer thin film bumps helped to improve the energy conversion efficiency by guiding more photons into the active semiconductor layers for further capture and absorption. The prepared homeotropic thin film precursor solution could be uniformly coated on photovoltaic cells by spin-coating techniques, and thus a massive and rapid manufacturing process for a large surface area could be developed. It has been shown that the patterned homeotropic thin-film-coated silicon photovoltaic cell samples improved the effective angle of incident sunlight. The energy conversion efficiency at the inclined incoming angle of 45° could be improved by up to 15%. The costly sunlight tracking system could be eliminated, and the strong acid/alkaline chemical texturing could be avoided. The idea of developing a cell with a higher energy conversion efficiency using more environmentally friendly technologies would thus become realized. The successful development of the new patterned liquid crystal polymer thin film system could pave the way for new designs for all types of photovoltaic cells.

## Figures and Tables

**Table 1:** Solar cell characteristic parameters at the various polymer precursor concentrations.

Concentration (%)	V_OC_ (V)	J_SC_ (mA/cm^2^)	FF (%)	Eff (%)
1	0.58	30.26	85.8	10.40
3	0.58	30.62	85.2	10.45
5	0.58	30.70	85.5	10.51
7	0.58	30.57	85.2	10.43
9	0.58	30.55	85.4	10.45
Control	0.58	29.86	85.7	10.25

## Data Availability

The original contributions presented in the study are included in the article and further inquiries can be directed to the corresponding author/s.
